# Cocoa tea (Camellia *ptilophylla*) water extract inhibits adipocyte differentiation in mouse 3T3-L1 preadipocytes

**DOI:** 10.1038/srep20172

**Published:** 2016-02-01

**Authors:** Kai Kai Li, Chuek Lun Liu, Hoi Ting Shiu, Hing Lok Wong, Wing Sum Siu, Cheng Zhang, Xiao Qiang Han, Chuang Xing Ye, Ping Chung Leung, Chun Hay Ko

**Affiliations:** 1 Institute of Chinese Medicine, The Chinese University of Hong Kong, Shatin, New Territories, Hong Kong SAR, China; 2 State Key Laboratory of Phytochemistry and Plant Resources in West China, The Chinese University of Hong Kong, Shatin, New Territories, Hong Kong SAR, China; 3 Shenzhen Research Institute, The Chinese University of Hong Kong, Shenzhen, China; 4 Department of Biology, School of Life Sciences, Sun Yat-sen University, Guangzhou 510275, China

## Abstract

Cocoa tea (*Camellia ptilophylla*) is a naturally decaffeinated tea plant. Previously we found that cocoa tea demonstrated a beneficial effect against high-fat diet induced obesity, hepatic steatosis, and hyperlipidemia in mice. The present study aimed to investigate the anti-adipogenic effect of cocoa tea *in vitro* using preadipocytes 3T3-L1. Adipogenic differentiation was confirmed by Oil Red O stain, qPCR and Western blot. Our results demonstrated that cocoa tea significantly inhibited triglyceride accumulation in mature adipocytes in a dose-dependent manner. Cocoa tea was shown to suppress the expressions of key adipogenic transcription factors, including peroxisome proliferator-activated receptor gamma (PPAR γ) and CCAAT/enhancer binding protein (C/EBP α). The tea extract was subsequently found to reduce the expressions of adipocyte-specific genes such as sterol regulatory element binding transcription factor 1c (SREBP-1c), fatty acid synthase (FAS), Acetyl-CoA carboxylase (ACC), fatty acid translocase (FAT) and stearoylcoenzyme A desaturase-1 (SCD-1). In addition, JNK, ERK and p38 phosphorylation were inhibited during cocoa tea inhibition of 3T3-L1 adipogenic differentiation. Taken together, this is the first study that demonstrates cocoa tea has the capacity to suppress adipogenesis in pre-adipocyte 3T3-L1 similar to traditional green tea

Obesity is one of the most common metabolic diseases worldwide^1^. Excessive accumulation of body fat may have an adverse effect on health, leading to reduced life expectancy and increased health problems, for instance, hypertension, type II diabetes mellitus, cardiovascular disease, cancer, and osteoarthritis^2^,^3^. At cellular level, obesity is-characterized by an increase in the number and size of adipocytes, which are differentiated from fibroblastic preadipocytes in adipose tissue^4^. Mouse 3T3-L1 preadipocytes have been extensively employed to study the cellular and molecular mechanisms of adipocyte differentiation^5^. Adipogenesis which involves preadipocyte proliferation and adipocyte maturation is driven by two major adipogenic transcription factors, C/EBP α and PPAR γ. These two transcription factors are known to coordinately activate adipogenesis hence are believed to play pivotal roles in modulating the entire differentiation process^6^,^7^. Upon activation, C/EBP α and PPAR γ cross-regulate each other to maintain high levels of expression. ; They also induce the expression of adipocyte-related proteins including SREBP-1c, FAT, FAS, ACC and SCD-1^8^,^9^,^10^.

Inhibition of adipocyte differentiation presents a key strategy to control obesity since an increase in adipose mass is caused by both adipocyte hypertrophy and adipocyte hyperplasia^11^. The use of herbal extract as a health supplement for anti-adipogenesis is increasingly popular in the past decades. A number of phytochemicals, including green tea polyphenols, resveratrol, curcumin, and proanthocyanidins have demonstrated anti-adipogenic potencies *in vitro* and *in vivo*^5^,^12^,^13^,^14^. Green tea (*Camellia sinensis*) is one of the most popular herbal supplements in the world^15^. It is well documented that green tea extract exhibits anti-adipogenic, hypolipidemic and hypoglycemic properties, Hence, it is widely used for multiple health benefits. On the contrary, most types of green tea contain high levels of caffeine, suggesting it may not be suitable for individuals with caffeine sensitivity In addition, there are several negative effects of caffeine on human behaviors including sleep deprivation^16^,^17^. Many researchers have attempted to remove caffeine from tea by organic solvent extraction and activated carbon adsorption^18^. However, the outcomes are rather undesirable due to high costs (for solvent and equipment) and loss of tea favor^19^.

Cocoa tea (*Camellia ptilophylla*), which belongs to the genus *Camellia*, is a naturally decaffeinated tea plant. It has been widely consumed by local inhabitants in the Longmen area of Guangdong Province in China^20^. It contains theobromine instead of caffeine, whereas the major catechin is (-)-gallocatechin gallate (GCG). Earlier work suggested that cocoa tea exhibited a profound cytotoxic effect on various cancer cell lines including HeLa, CNE2, and MGC-803, and HepG2^21^,^22^. More recently, we showed that the administration of cocoa tea in high-fat diet-induced obesity mice resulted in dose-dependent reduction in body weight, fat pad mass, liver weight, total liver lipid, liver triglyceride and cholesterol, as well as plasma lipids (triglyceride and cholesterol)^23^. However, little is known about how cocoa tea influences adipogenesis and lipogenesis in adipocytes. Based on our *in vivo* findings, we hypothesized that cocoa tea could directly inhibit the differentiation of preadipocytes to adipocytes. Hence, in order to investigate the unclear underlying mechanisms of the biological effects of cocoa tea extract in adipocyte metabolism, we carried out the present study using the cell line 3T3-L1 preadipocyte with the same species with *in vivo* study to elucidate the anti-adipogenic effects of cocoa tea. Many reports suggest that mouse 3T3-L1 is one of the most well characterized and reliable *in vitro* models for studying the commitment of preadipocytes to differentiation into adipocytes. This cell line possesses some phase 2 drug metabolizing enzymes, including ATP-Binding Cassette, Sub-Family C member 1, ATP-Binding Cassette, Sub-Family C member 4, Glutathione S-transferase A2, and Glutamate-cysteine ligase catalytic subunit^24^. In the present study, we used mouse 3T3-L1 preadipocyte cell line as a model to investigate the anti-adipogenic potential of cocoa tea and its underlying mechanism in modulating adipocyte metabolism.

## Results

### HPLC analysis

HPLC chemical profile of cocoa tea extract (CTE) was different from green tea extract (GTE) (See Supplementary Information). Cocoa tea contains theobromine (8.43 ± 0.51%) and GCG (10.78 ± 0.63%) as the main alkaloid and catechin, respectively. For traditional green tea, the main alkaloid was caffeine (6.12 ± 0.03%) while the major catechin was EGCG (8.54 ± 0.09%). Particularly, the relative compositions of catechins in cocoa tea increased in the order: ECG < EGCG < C < GCG. Whereas the relative compositions of catechins in green tea extract increased in the order: ECG < EC < EGC < EGCG. In addition, the water extract of cocoa tea also demonstrated the presence of a proanthocyanidin, GC-(4 → 8)-GCG (0.94 ± 0.28%). No caffeine content was found in cocoa tea.

### Effect of CTE on 3T3-L1 cell viability

**Figure 1:**
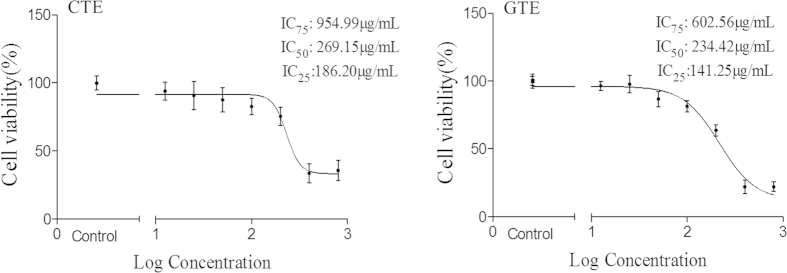
Effect of CTE and GTE on cell viability in 3T3-L1 cells. Cell viability was determined by MTT assay. CTE: Cocoa tea extract; GTE: Green tea extract.

The cytotoxic effects of CTE on cell viability were studied using MTT assay. As shown in Fig. 1, both of CTE and GTE had cytotoxic effect at high doses. Treatment of CTE and GTE for 48 hour (h) reduced preadipocyte proliferation and viability (Fig. 1)., At 200 μg/ml, both CTE and GTE significantly decreased the cell viability (*p* < 0.01), resulting in an IC_50_ value of 269.15 μg/ml and 234.42 μg/ml, respectively.

### Effects of CTE on adipogenic differentiation

**Figure 2:**
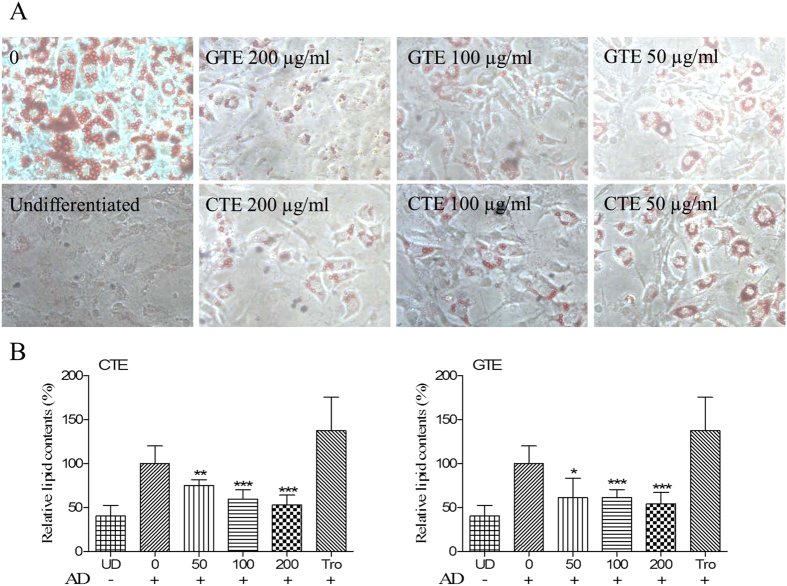
Anti-adipogenic effects of CTE and GTE on 3T3-L1 cells. (**A**) Effects of CTE and GTE on the differentiation of 3T3-L1 cells. 3T3-L1 cells were cultured in adipocyte differentiation cocktail media with or without the treatment of CTE or GTE, After 8 days culture, the cells were stained with Oil-red O, and then photographed under the microscope (Magnification: 40 × ), (**B**) The relative lipid content in different treatment groups. CTE: cocoa tea extract; GTE: green tea extract. Tro: troglitazone. The results are presented as the mean ± SD of triplicate tests. Significant difference: *p < 0.05, **p < 0.01, ***p < 0.001 as compared to control group.

To investigate the anti-adipogenic effect of CTE, intracellular lipid accumulation was determined in mature adipocytes. Adipogenic differentiation of 3T3-L1 preadipocytes was examined on day 8 by Oil-Red O staining (Fig. 2A). Mature adipocytes were identified and characterized by a quantity of oil droplets in the cells which are not seen in undifferentiated cells. Based on microscopic observation, it showed that 3T3-L1 cells which were treated with CTE and GTE maintained the fibroblastic shape and contained less lipid droplets. At 50 μg/ml, CTE and GTE significantly reduced the formation of oil droplets to 75.08 ± 6.68% and 71.23 ± 13.14% compared to mature adipocytes (*p* < 0.01 and *p* < 0.05, respectively) (Fig. 2B).

**Figure 3:**
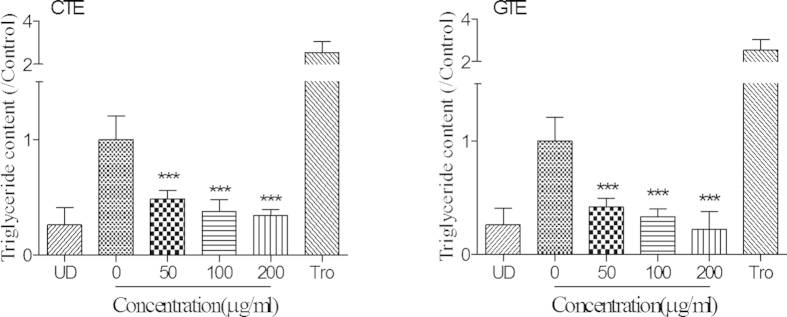
Effect of CTE and GTE on the triglyceride deposition in differentiated 3T3-L1 cells. 3T3-L1 cells were cultured in adipocyte differentiation cocktail media with or without the treatment of CTE or GTE. CTE: Cocoa tea extract; GTE: green tea extract. The results are presented as the mean ± SD of triplicate tests. Significant difference: ***p < 0.001 as compared to control group.

The intracellular content of triglycerides (TG) was also quantified on day 8 of adipogenic differentiation. The treatment with CTE and GTE dramatically reduced lipid accumulation in cells. Whereas, the treatment with 3-isobutyl-1-methylxanthine (IBMX), Dexamethasone and Insulin (MDI) alone was found to significantly increase TG content, in the CTE and GTE treatment groups (See Fig. 3). TG content was significantly decreased to 30% and 22% by CTE and GTE at 200 μg/ml, respectively (*p* < 0.001). In addition, CTE and GTE were found to reduce intracellular TG content in a concentration dependent manner. There was no significant difference between CTE and GTE treated cells. Furthermore, a considerable higher amount of TG was found in the differentiation group compared to the group treated with 200 μg/ml of GTE. This might be due to the fact that differentiating cells can produce a trace amount of TG. Secondly, 200 μg/ml of GTE and CTE significantly decreased the viability of 3T3-L1 cells as shown in our cytotoxicity test. Taken together, our results from Oil Red staining and triglyceride assay demonstrated that CTE and GTE inhibited the adipogenic differentiation of 3T3-L1 cells.

### Effect of CTE on the expressions of adipogenic transcription factors

**Figure 4:**
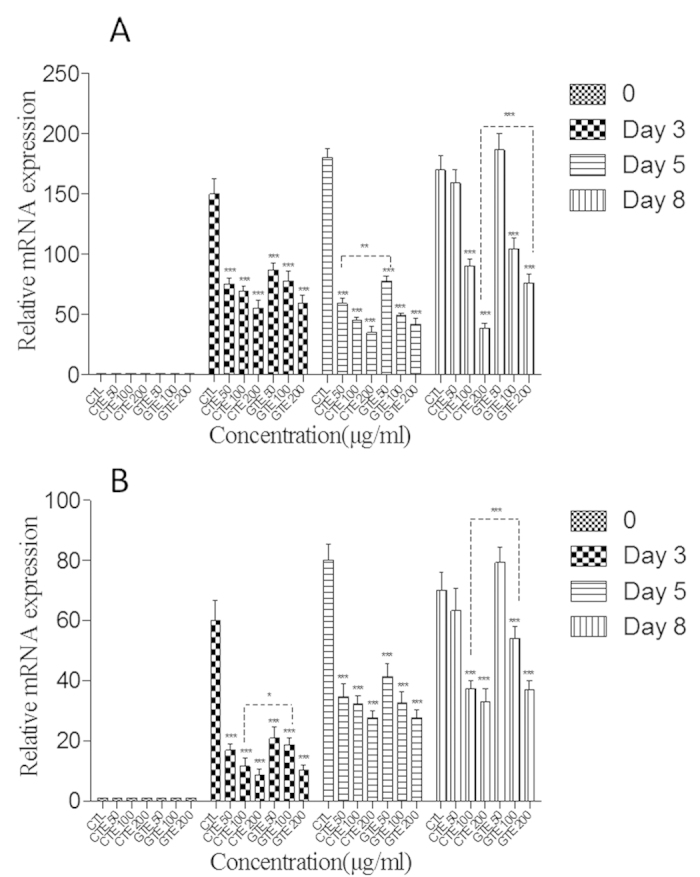
Effect of CTE and GTE on the gene expressions of the key adipogenic transcription factors, PPAR γ and C/EBP α associated with (A) PPAR γ; (B) C/EBP α; CTE: cocoa tea extract; GTE: green tea extract. All values are expressed as mean ± SEM of triplicate tests. *p < 0.05; **p < 0.01; ***p < 0.001 as compared to control group.

**Figure 5:**
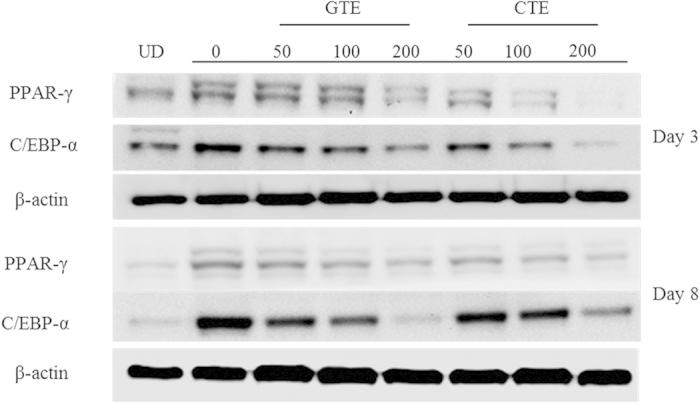
Effect of CTE and GTE on the protein expressions of the key adipogenic transcription factors, PPAR γ and C/EBP α on day 3 and 8. The cells were induced to differentiate into adipocytes in MDI medium with or without CTE and GTE. The experiment was performed three to four times using independently prepared cell lysates, and representative blots were shown.

Adipogenic differentiation with lipid accumulation is accompanied by the expressions of master adipogenic transcription factors in preadipocytes, such as C/EBP α and PPAR γ. We next investigated whether CTE and GTE influence the expressions of PPAR γ and C/EBP α during adipogenic differentiation. As shown in Fig. 4, MDI significantly induced the expression of PPAR γ and C/EBP α. Compared with the control group (MDI), CTE and GTE significantly suppressed the expressions of C/EBP α and PPAR γ. For PPAR γ, the inhibitory effect of CTE was stronger than that of GTE at same concentration on day 5 and day 8 (*p* < 0.05). For C/EBP α, the inhibitory effect of CTE at 100 μg/ml was stronger than that of GTE on both day 3 and day 8 (*p* < 0.05). Analysis of the protein levels of these transcription factors on day 3 and day 8 showed that both CTE and GTE treatments significantly decreased the expressions of PPAR γ and C/EBP α, which was consistent with our mRNA expression results. As shown in Fig. 5, the expressions of PPAR γ and C/EBP α were increased significantly in the control group, when compared with undifferentiated group (UD), at day 8. Although low dose (50 μg/ml) of GTE showed no significant inhibition on the expression of PPAR γ and C/EBP α, GTE could inhibit the protein levels of PPAR γ and C/EBP α at doses of 100 μg/ml and 200 μg/ml (p < 0.05). Similarly, CTE could dose-dependently inhibit PPAR γ expression. However, CTE only inhibited C/EBPα at 200 μg/ml (p < 0.05).

### Effects of CTE on the expressions of adipocyte-specific genes

**Figure 6:**
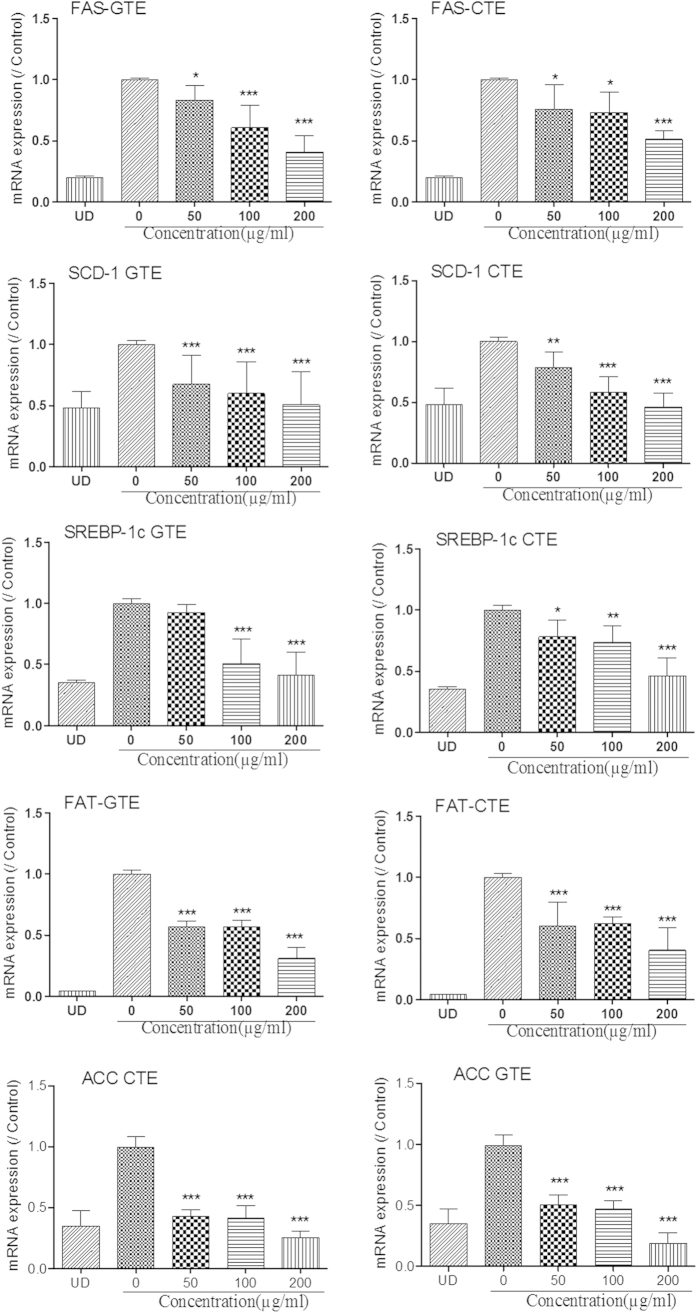
Effect of CTE and GTE on gene expressions of the adipocyte-specific genes of 3T3-L1 adipocyte differentiation. The cells were induced to differentiate into adipocytes in MDI medium with or without CTE and GTE. mRNA were extracted and the expression of SREBP-1c, FAS, ACC, FAT and SCD-1 genes were detected using one-step RT-PCR. CTE: cocoa tea extract; GTE: green tea extract. Each value represents the Mean ± SEM of triplicate test. *p < 0.05; **p < 0.01; ***p < 0.001 as compared to control group.

**Figure 7:**
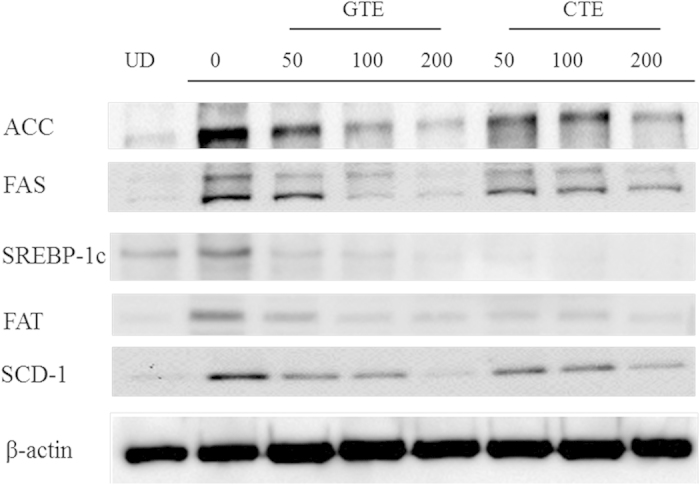
Cocoa tea inhibits the protein expressions of the adipocyte-specific genes of 3T3-L1 adipocyte differentiation. The cells were induced to differentiate into adipocytes in MDI medium with or without CTE and GTE. The proteins were extracted and the expressions of SREBP-1c, FAS, ACC, FAT and SCD-1 genes were detected using Western blot assay. The western blot was performed three to four times using independently prepared cell lysates, and representative blots were shown.

*In vitro* differentiation of adipocyte is completed about one week after the initiation of MDI treatment. During differentiation, a series of key molecular events occur which determines the phenotype of adipocytes. Next, we investigated the effect of CTE on the expression of the adipocyte-specific genes such as FAS, SCD-1, FAT, ACC and SREBP-1c. As shown in Fig. 6 and Fig. 7, MDI significantly induced the mRNA and protein expressions of FAS, SCD-1, FAT, ACC andSREBP-1c.The increase in the mRNA levels of SREBP-1c, SCD-1, ACC, FAT, and FAS in 3T3-L1 cells were reversed by CTE and GTE. Treatments with CTE and GTE significantly decreased the protein levels of FAS, SCD-1, FAT, ACC and SREBP-1c, compared to those of positive control using mature adipocytes (Fig. 7). The expressions of these adipocyte-specific genes showed no significant difference between CTE and GTE treated adipocytes. These findings suggested that CTE strongly suppressed the adipogenic differentiation of 3T3-L1 preadipocytes by decreasing the expression of adipocyte-specific genes.

### Effect of CTE on MAPKs phosphorylation

The MAPK pathways play a crucial role in signaling the gene expressions of C/EBP α and PPAR γ. To elucidate the effect of CTE on MAPK pathway, phosphorylations of ERK, JNK, and p38 were examined. Our results indicated that MDI treatment led to increase of all MAPKs in 15 mins (for ERK and P38) or 30 mins (for JNK), followed by decreasing trend. Our data indicated that that the phosphorylation of ERK was significantly decreased 2 hours after MDI treatment, which in line to some reports. Phosphorylation of JNK and P38 may be long acting to the cells, therefore the decreases were gradual and insignificant in 2 hours. Some reports suggested that the phosphorylation of MAPK kinases were decreased even longer, i.e. at 4 hours, 8 hours but without significant changes at 2 hours after MDI treatment, and our findings for JNK and P38 were somehow similar. CTE treatment markedly decrease the levels of MDI-induced phosphorylations of ERK, p38 and JNK.

## Discussion

It has been suggested that adiposity is reduced by the inhibition of adipogenesis, which is associated with reductions of number and lipid content of adipocytes^25^. There are many therapeutic strategies for treating obesity, including balance in energy intake and expenditure, suppression in preadipocyte differentiation and lipogenesis, as well as inductions of lipolysis and adipocyte apoptosis^26^. Our previous study indicated that cocoa tea has a beneficial effect against high-fat diet induced obesity, hepatic steatosis, and hyperlipidemia in mice^23^. In this study, we demonstrated that the addition of CTE to 3T3-L1 cells significantly reduced lipid droplet accumulation, triglyceride content, as well as the expressions of key adipogenic transcription factors and adipocyte-specific genes.

The beneficial effect of dietary green tea on controlling plasma and hepatic lipid levels have been well documented in various animal studies^27^. Positive findings from these pre-clinical studies have also been confirmed by a number of human clinical trials, indicating the anti-adipogenic effect of green tea^28^,^29^. The tea catechin, particularly EGCG, has been demonstrated to reduce body weight in animal models of obesity. Many mechanisms have been proposed for the anti-adipogenic activity of EGCG. These include suppression of preadipocyte survival and proliferation, induction of cell apoptosis^30^,^31^, activation of AMP-activated protein kinase (AMPK)^32^, and modulation of the expression of adipocyte-related genes C/EBP α, PPAR γ^33^,^34^,^35^. These suggestions were consistent with our findings that green tea inhibits adipocyte formation. Kim and Sakamoto (2012) reported that EGCG suppressed clonal expansion of differentiating adipocytes by reducing the transcriptional activity of FoxO1 via the PI3K/Akt and MEK/ERK pathways^14^. Ku *et al.* (2012) showed that EGCG suppressed IGF-I and IGF-II signaling in preadipocyte mitogenesis via 67LR but not AMPK pathway^12^. Furthermore, some reports showed that the caffeine inhibited the adipogenic differentiation and reduced the body weight and adipose tissue weight in animal models^36^,^37^,^38^,^39^.

Cocoa tea is a naturally decaffeinated tea. It is believed that the main components of cocoa tea, theobromine and GCG, play central roles in its anti-adipogenic effect. The stimulant effect of theobromine is 20% of that of caffeine^40^. Previous work found that GCG and theobromine significantly reduced the plasma cholesterol and triglycerides^41^,^42^,^43^,^44^. Jang *et al.* (2015) recently found that theobromine inhibited adipogenic differentiation of 3T3-L1 preadipocytes during early stages of adipogenesis by regulating the expressions of C/EBP α and PPAR γ through AMPK and ERK/JNK signalling pathways^45^. Given that cocoa tea has a high content of theobromine, cocoa tea appears as a good source for theobromine supplement. However, theobromine dissolves poorly in normal pH and water temperature, thus limiting its applications (European Pharmacopoeia 5.0 (2005) Monographs: 0298 Theobromine: 2554). Kurihara *et al.* (2006) found that cocoa tea reduced the lymphatic absorption of TG^46^. Our previously study found that cocoa tea had a relieving effect on obesity, hepatomegaly, hepatic steatosis and elevated plasma lipid levels in mice fed with high-fat diet^23^. Therefore, cocoa tea might be an alternative for people who are intolerant of green tea due to its high caffeine content. In our present study, we aimed to investigate the anti-adipogenic activity of cocoa tea and its underlying mechanism.

Obesity is caused by excessive growth of adipose tissue mass as a result of increase in number and size of adipocytes differentiated from preadipocytes^47^,^48^,^49^. Our results showed that CTE and GTE at high dose significantly inhibited the viability of 3T3-L1 cells. They also remarkably suppressed the adipogenic differentiation in a concentration-dependent manner, as indicated by less mature adipocytes withoil droplets and reduced level of accumulated intracellular triglyceride. These results suggest that CTE inhibits adipogenesis and accumulation of lipid droplets during the differentiation of 3T3-L1 cells. This is also consistent with our previous results in mice that cocoa tea treatment reduced liver cholesterol at both 2% and 4% (55.9 ± 3.2%, *p* < 0.001, and 72.1 ± 1.6%, *p* < 0.001)^23^. Taken together, these findings suggested that CTE had an inhibitory role in the conversion of 3T3-L1 cells during adipogenesis, apart from its inhibitory effect on cell viability which is similar to that of GTE.

Adipogenesis is a complex process which is tightly regulated by sequential activations of various transcriptional factors. Preadipocyte culture systems were well established for studying cellular and molecular mechanisms of adipocyte differentiation. Adipokines, such as C/EBP α and PPAR γ, are some of the most important genes during adipogenesis. They have a direct impact on the development of fat cells^50^,^51^,^52^. The expressions of both C/EBP α and PPAR γ increased dramatically from undetectable levels in preadipocytes to detectable levels and full expressions two days and 5 days after the induction of differentiation, respectively^53^. In our present study, we investigated the effect of cocoa tea extract on the expression of PPAR γ, C/EBP α on day 3 (early stage), day 5 (intermediate stage) and day 8 (final stage) during adipogenic differentiation. The reduced expression in protein levels of PPAR γ and C/EBP α on day 3 and 8 is also in accordance with our suggestion. An extension of this study is to apply the inhibitor(s) and enhancers(s) of PPAR γ, C/EBP α and also gene-knockout technology to further verify our findings. Similar to the bioactivity of green tea and EGCG, cocoa tea also inhibit the adipogenic differentiation of 3T3-L1, which is likely to be mediated by down-regulating the expressions of C/EBP α and PPAR γ^42^,^43^.

C/EBP α and PPAR γ are known to induce the expressions of adipocyte-specific genes which control fatty acid metabolism in adipocyte. These genes include fatty acid binding protein (aP2), FAS, SCD-1, FAT and lipoprotein lipase (LPL)^54^,^55^,^56^. FAS and SCD-1 regulate the expression of genes involved in lipogenesis and fatty acid desaturation^57^. ACC catalyzes the synthesis of malonyl-CoA, a metabolite that plays a pivotal role in the synthesis of fatty acids as the donor of “C2 units”. Hence we investigated the effect of cocoa tea extract on the expression of these adipocyte-specific genes in mature adipocytes. We found that CTE reduced mRNA and protein expressions of FAS and ACC, which are involved in the late stage of adipogenesis (See Figs 6 and 7). Furthermore, gene and protein expressions of FAT and SCD-1 were also reduced by cocoa tea treatment (See Figs 6 and 7). SREBP-1c is found to be a crucial transcriptional regulator involved in adipogenesis. It is associated with the production of an endogenous PPAR γ ligand which reinforces PPAR γ activity^58^. In our present study, we found that CTE significantly inhibited the expression of SREBP-1c. Taken together, it was suggested that the anti-adipogenic effect of cocoa tea was mediated by as of anti-adipogenic effect of traditional green tea down-regulation of the expressions of transcription factors such as PPAR γ, CEBP α and SREBP1c during adipocyte differentiation. This is also consistent with our previous findings that cocoa tea treatment reduced the level of PPAR γ dose-dependently in a high-fat induced obesity mice model^23^.

**Figure 8:**
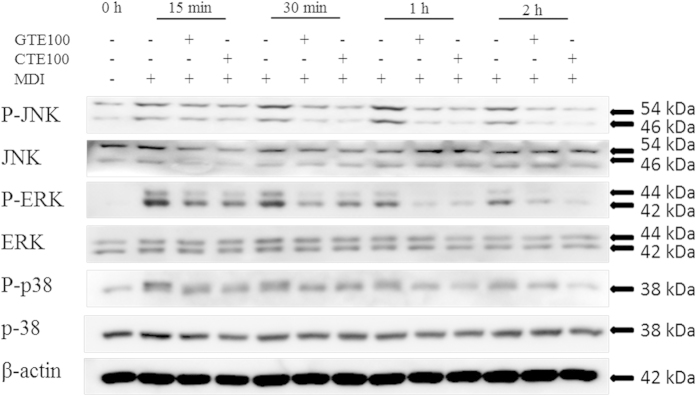
Cocoa tea inhibits JNK, ERK and p38 phosphorylation during its inhibition of 3T3-L1 adipocyte differentiation. The cells were induced to differentiate into adipocytes in MDI medium with or without CTE OR GTE for 15 min, 30 min, 1 h and 2 h, the protein were extracted and the phosphorylation of JNK, ERK and p38 were detected using Western Blot. Immunoblotting was performed three to four times using independently prepared cell lysates, and representative blots were shown.

The extracellular signal-regulated kinases (ERK1/2), Jun amino terminal kinases (JNK), and p38 mitogen-activated protein kinases (p38) are the members of mitogen-activated protein kinases (MAPKs) that play a pivotal part in many essential cellular processes, including cell proliferation and adipocytes differentiation. Proteins of the MAPKs second messenger pathway, specifically pro-adipogenic ERK and anti-adipogenic p38 MAPK^59^,^60^, have been implicated to regulate preprogrammed adipocyte differentiation. The ERK pathway interacts primarily with mitogens and growth factors. It plays a key role in cell proliferation, survival, and differentiation^61^. It was reported that reduction of ERK1/2 activation in preadipocytes suppressed adipocytes differentiation^62^,^63^,^64^,^65^. Moreover, the MAPK signaling pathway regulates the expression of C/EBP α and PPAR γ mRNA during adipogenesis in 3T3-L1 cells^66^. We found that CTE inhibited the phosphorylations of ERK, p38 and JNK in a concentration dependent manner, similar to those observed with GTE. Thus, it is speculated that CTE induces apoptosis and inhibits adipogenesis in 3T3-L1 cell via suppression of ERK1/2, p38 and JNK phosphorylation which is partly dependent on MAPK kinase/ERK signaling pathway (See Fig. 8).

Little is known about the pharmacokinetic of cocoa tea. The chemical compositions of cocoa tea are very similar to traditional green tea, except the contents of theobromine and GCG. Oral absorption of theobromine from the digestive tract is slower (an estimated peak plasma time of 2.5 h) compared with caffeine (0.5 h)^67^. Recently, a double-blind study in human of Hodgson *et al.*^68^ measured the transient changes in total and free concentrations of catechins in plasma from healthy males following the consumption of a single green tea extract dose (559.2 mg total catechins, 120.4 mg caffeine). They found that the ratios of the maximum concentrations in plasma to the concentrations in the tradition green tea supplement relative to the total catechins, respectively, GCG was higher than EGCG. 22% of GCG existed in free form, while non-gallated catechins EGC, EC, and C mainly present in the conjugated form. Further investigations are needed to study the pharmacokinetic of high content of GCG-containing cocoa tea, instead of EGCG predominant green tea.

In summary, our findings suggested that cocoa tea has the potential to inhibit the cell viability and adipogenic differentiation of 3T3-L1 cells. To our best knowledge, this is the first study which demonstrates that cocoa tea exhibits attenuation of molecular events in adipogenesis in 3T3-L1 preadipocytes. We proposed that the underlying mechanisms of the anti-adipogenic activity of cocoa tea were similar to that of traditional green tea. These further suggested that cocoa tea might be able to inhibit obesity. In the present study, we aimed to study the direct effect and its molecular events of cocoa tea in adipogenesis using one of the key players, the preadipocytes. Future studies will include investigations of the bioavailability and systemic effect of cocoa tea, the interactions among immune cells, adipocytes cytokines and growth factors regulating adipogenesis, as well as the pharmacokinetic studies of cocoa tea in *in vivo* disease models. In addition, the molecular mechanism of how cocoa tea and its main chemical components such as theobromine and/or GCG exerted its anti-adipogenic effect remain to be further defined.

## Methods

### Herbal extraction and HPLC analysis of tea aqueous extract

Green tea leaves were purchased from renowned supplier in Guangdong Province, China. Cocoa tea leaves were obtained from the Tea Research Institute of Guangdong Province. The extraction protocol was used as described previously^23^. High-performance liquid chromatography (HPLC) analysis was performed using Agilent 1100 series HPLC System, equipped with G1329A ALS Auto-sampler and G1315A Diode Array Detector (Agilent Technologies, USA). Sample solution was injected onto a Supelco Discovery RP Amide C16 guard column (15 cm × 4.6 mm, 5 μm) (Sigma-Aldrich, Inc., USA). A gradient elution was carried out using the following solvent systems: mobile phase A-0.05%- phosphoric acid; mobile phase B-acetonitrile. The elution was performed with a gradient procedure as follows: 0-1 min, 2% B; 2–30 min, from 2% B to 50% B. The sample injection volume was 10μl. Elution was performed at a solvent flow rate of 0.8 ml/min. A standard mixture which contains theanine (Thea), theobromine (TB), caffeine (CAF), theacrine (TC), epigallocatechin (EGC), catechin (C), epicatechin (EC), epigallocatechin gallate (EGCG), gallocatechin (GC), gallocatechin gallate (GCG), and epicatechin gallate (ECG) in methanol was prepared and analyzed. Purine alkaloid and catechin compounds were identified by comparing the retention time and spectral data with those of authentic standards. All analyses were repeated three times.

### Cell Culture

Mouse 3T3-L1 preadipocytes (American Type Culture Collection (ATCC)) were cultured in 12 well culture plates with complete medium (high glucose Dulbecco’s Modified Eagle Medium (Gibco), 10% fetal bovine serum (FBS) and 50 μg/ml penicillin/streptomycin). Upon confluence, the media were changed the following day and replaced with adipogenic differentiation cocktail media containing 0.5 mM 3-isobutyl-1-methylxanthine (IBMX), 1 mM dexamethasone (Sigma, USA) and various concentrations of CTE and GTE. The culture was further incubated at 37 °C, 5%CO_2_ for two days. After that, the media were replaced with DMEM supplemented with 10% fetal bovine serum (FBS) and insulin (10 μg/mL) for 2 days. The extent of differentiation was determined on day 8.

### *In vitro* cytotoxicity assay

Mouse 3T3-L1 preadipocytes were seeded in a 96-well plate (5 × 10^3^ cells/well) and incubated at 37 °C, 5%CO_2_ with complete medium for 24 h. After 24 h, the medium was changed to complete medium supplemented with CTE or GTE at various concentrations for 48 h. MTT solution (5 mg/ml in PBS) was then added to the plate (20 μl/100 μl medium/well) and incubated for 4 h at 37 °C. The resultant formazan product was dissolved in DMSO (200 μl /well) and measured at 492 nm by a microplate spectrophotometer.

### Oil Red O Staining

To investigate the effect of CTE on lipid accumulation in 3T3-L1 preadipocytes, the cells were differentiated in the presence of CTE or GTE at various concentrations. Troglitazone, a known PPAR γ agonist, was used as a positive control. Intracellular lipid accumulation was determined by Oil red O staining on day 8. After removal of culture media, the cells were washed twice with PBS, fixed with 10% Formalin, and stained with Oil red O (six parts 0.6% Oil red O dye in isopropanol and four parts water) for 30 min. After rinsing three times with distilled water, the cells were photographed under microscope. To quantify the lipid accumulation, lipid and Oil red O were dissolved in isopropanol and absorbance was measured by a microplate spectrophotometer at 495 nm. The percentage of oil red O stained material relative to control wells was calculated as 495 nm (tea water extracts)/495 nm (control) × 100.

### Triglyceride Content

To analyze the intracellular content of triglycerides (TG), the cells were washed with PBS, harvested by trypsinisation and then resuspended in 1 ml PBS. The cell suspension was homogenized by sonication for 5 min^10^. Triglyceride content was determined using a commercial triglyceride assay kit according to the manufacturer’s protocol (GPO-PAP, Roche diagnostics, Mannheim, Germany). The protein concentration was determined using a Bradford reagent (Sigma, St. Louis, MO).

### Quantitative Real-Time PCR Analysis

Total RNA was extracted from 3T3-L1 cells at desired time points (Day 3, 5 and 8) after adipogenic differentiation with various concentrations of CTE and GTE. Selected genes were amplified and quantified by a one-step PCR using a Quantifast SYBR Green RT-PCR kit. The sequences of primers used for quantitative real-time PCR were showed in Table 1. PCR conditions were as follow: 1 cycle of 50 °C for 10 min and 95 °C for 5 min, 49 cycles of 95 °C for 10 s, 60 °C for 30 s and 72 °C for 30 s, followed by 1 cycle of 95 °C for 1 min. Relative gene expression was expressed as relative mRNA level compared with a control, was calculated after normalization to GAPDH following the 2−^ΔΔCT^ method. CT value was presented as mean of duplicate measurements.

### Protein extraction and Western Blot

The cells on day 3 and day 8 were collected and lysed in a RIPA buffer for 30 min on ice. The lysate was centrifuged at 14000 rpm for 15 min at 4 °C. Protein concentration was measured using a Bio-Rad Dc Protein Assay (Bio-Rad, Hercules, CA USA). Protein samples at same amount (40 μg) were separated on 8% SDS-polyacrylamide gel and electrophoretically transferred (100 V, 2 h) onto a nitrocellulose membrane (Pall Gelman Laboratory, Ann Arbor, MI USA). The membranes were blocked with 5% non-fat dry milk for 1 h, and incubated overnight at 4^o^C with primary antibodies. After washing, the membranes were incubated with the secondary horseradish peroxidase-conjugated antibodies (Invitrogen, Carlsbad, CA, USA) for 1 h. The protein of interest was identified using an enhanced chemiluminescence assay kit (GE Healthcare, UK).

### Statistical Analysis

All values are presented as Mean ± SD unless otherwise specified. The data was analyzed by one-way ANOVA using SPSS version 16.0. The differences compared with control group were assessed using Duncan’s multiple range tests. Statistical significance was considered at *p* < 0.05.

## Additional Information

**How to cite this article**: Li, K. K. *et al.* Cocoa tea (Camellia *ptilophylla*) water extract inhibits adipocyte differentiation in mouse 3T3-L1 preadipocytes. *Sci. Rep.* **6**, 20172; doi: 10.1038/srep20172 (2016).

## Supplementary Material

Supplementary Materials

## Figures and Tables

**Table 1:** Primer sequences used in RT-PCR analysis.

Gene name		Forward primer	Reverse primer
GAPDH*	NM_008084	AAGAAGGTGGTGAAGCAGGCATC	CGAAGGTGGAAGAGTGGGAGTTG
PPAR γ	NM_011146	TTCAGCTCTGGGATGACCTT	CGAAGTTGGTGGGCCAGAAT
C/EBP a	NM_007678	GTGTGCACGTCTATGCTAAACCA	GCCGTTAGTGAAGAGTCTCAGTTTG
ACC	NM_133360	GCGTCGGGTAGATCCAGTT	CTCAGTGGGGCTTAGCTCTG
FAS	NM_007988	TTGCTGGCACTACAGAATGC	AACAGCCTCAGAGCGACAAT
SCD1	NM_009127	CATCGCCTGCTCTACCCTTT	GAACTGCGCTTGGAAACCTG
FAT	NM_007643	TAGTAGAACCGGGCCACGTA	CAGTTCCGATCACAGCCCAT
SREBP-1c	NM_011480	ATCGCAAACAAGCTGACCTG	AGATCCAGGTTTGAGGTGGG

PPAR γ, peroxisome proliferator-activated receptor γ C/EBP a, CCAAT/enhancer-binding protein a ACC, Acetyl-CoA carboxylase FAS, fatty acid synthase SCD1, stearoyl-CoA desaturase FAT, fatty acid translocase SREBP-1c, Sterol regulatory element binding transcription factor 1c.

^*^GAPDH: glyceraldehyde-3-phosphate dehydrogenase.
