# Supplementary material for: Cocoa tea (Camellia ptilophylla) water extract inhibits adipocyte differentiation in mouse 3T3-L1 preadipocytes
Source: Sci Rep. 2016 Feb 1;6:20172. doi: 10.1038/srep20172 (PMC4735603; doi:10.1038/srep20172)
Supplement: Supplementary Materials [file srep20172-s1.docx]

**Supplementary materials:**

**Title: Cocoa tea (Camellia ptilophylla) water extract inhibits adipocyte differentiation in mouse 3T3-L1 preadipocytes**

**Authors: Kai Kai Li, Chuek Lun Liu, Hoi Ting Shiu, Hing Lok Wong, Wing Sum Siu, Cheng Zhang, Xiao Qiang Han, Chuang Xing Ye, Ping Chung Leung, Chun Hay Ko**


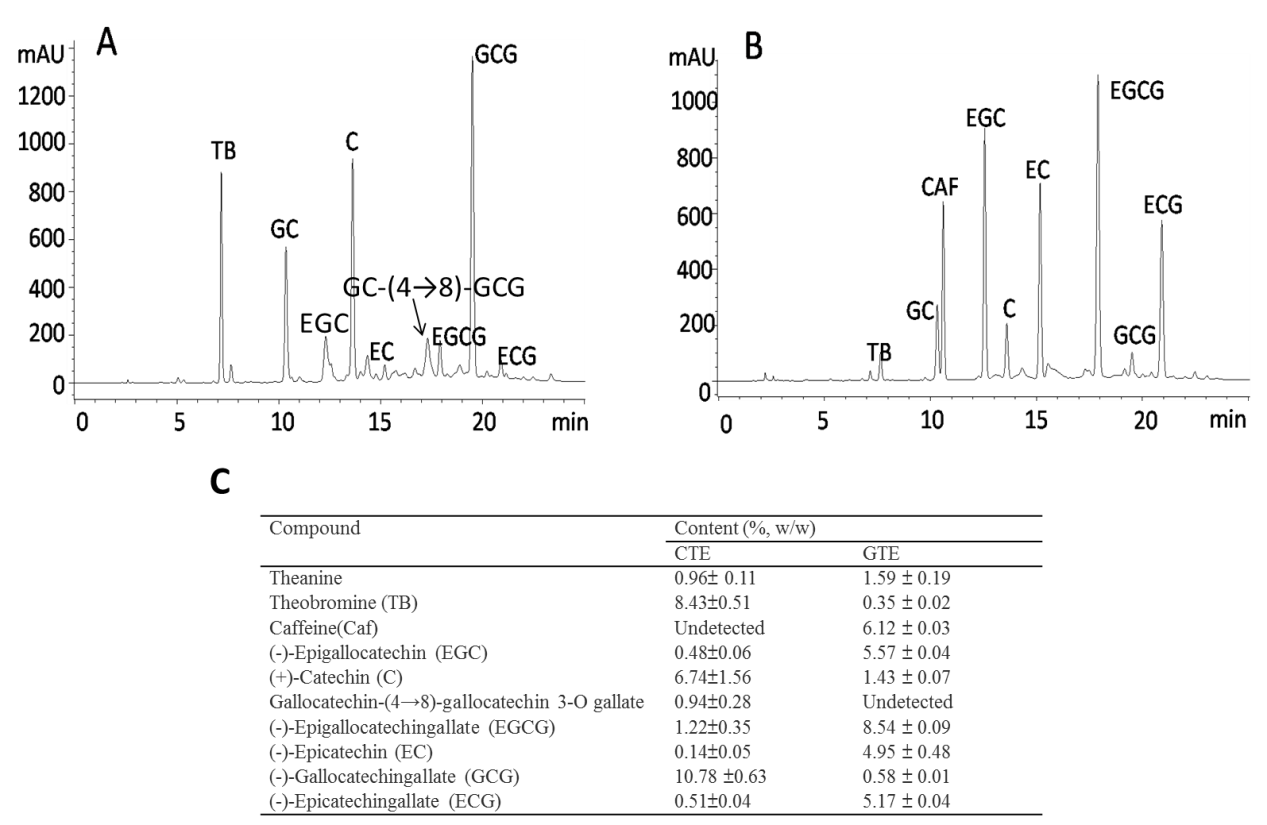


Fig 1. The alkaloids and catechins contents in cocoa tea water extract (CTE) and green tea water extract (GTE). Detection was performed using Agilent 1100 series HPLC System with a Supelco Discovery RP Amide C16 guard column at UV 210 nm. HPLC chromatography of (A) CTE and (B) GTE are shown.

Table 1. Total alkaloids and catechins contents in CTE and GTE.

| Compound | Content (%, w/w) | |
| --- | --- | --- |
|  | Cocoa tea | Green tea |
| Theanine | 0.96± 0.11 | 1.59 ± 0.19 |
| Theobromine (TB) | 8.43±0.51 | 0.35 ± 0.02 |
| Caffeine(Caf) | Undetected | 6.12 ± 0.03 |
| (-)-Epigallocatechin (EGC) | 0.48±0.06 | 5.57 ± 0.04 |
| (+)-Catechin (C) | 6.74±1.56 | 1.43 ± 0.07 |
| Gallocatechin-(4→8)-Gallocatechin 3-O gallate (GC-(4→8)-GCG) | 0.94±0.28 | Undetected |
| (-)-Epigallocatechingallate (EGCG) | 1.22±0.35 | 8.54 ± 0.09 |
| (-)-Epicatechin (EC) | 0.14±0.05 | 4.95 ± 0.48 |
| (-)-Gallocatechingallate (GCG) | 10.78 ±0.63 | 0.58 ± 0.01 |
| (-)-Epicatechingallate (ECG) | 0.51±0.04 | 5.17 ± 0.04 |
